# Molecular Mechanisms Directing Migration and Retention of Natural Killer Cells in Human Tissues

**DOI:** 10.3389/fimmu.2018.02324

**Published:** 2018-10-11

**Authors:** Roberta Castriconi, Paolo Carrega, Alessandra Dondero, Francesca Bellora, Beatrice Casu, Stefano Regis, Guido Ferlazzo, Cristina Bottino

**Affiliations:** ^1^Dipartimento di Medicina Sperimentale, University of Genova, Genova, Italy; ^2^Centro di Eccellenza per la Ricerca Biomedica, University of Genova, Genova, Italy; ^3^Dipartimento di Patologia Umana, University of Messina, Messina, Italy; ^4^Istituto di ricovero e cura a carattere scientifico (IRCCS) Giannina Gaslini, Genova, Italy

**Keywords:** natural killer cells, chemokines and chemokine receptors, migration and residency, tumor and inflammation, pregnancy

## Abstract

A large body of data shows that Natural Killer (NK) cells are immune effectors exerting a potent cytolytic activity against tumors and virus infected cells. The discovery and characterization of several inhibitory and activating receptors unveiled most of the mechanisms allowing NK cells to spare healthy cells while selectively attacking abnormal tissues. Nevertheless, the mechanisms ruling NK cell subset recirculation among the different compartments of human body have only lately started to be investigated. This is particularly true for pathological settings such as tumors or infected tissues but also for para-physiological condition like pregnant human uterine mucosa. It is becoming evident that the microenvironment associated to a particular clinical condition can deeply influence the migratory capabilities of NK cells. In this review we describe the main mechanisms and stimuli known to regulate the expression of chemokine receptors and other molecules involved in NK cell homing to either normal or pathological/inflamed tissues, including tumors or organs such as lung and liver. We will also discuss the role played by the chemokine/chemokine receptor axes in the orchestration of physiological events such as NK cell differentiation, lymphoid organ retention/egress and recruitment to decidua during pregnancy.

## Introduction

The initial view describing Natural Killer (NK) cells as a quite homogeneous CD3^neg^ CD56^+^ circulating lymphocyte population has been largely overcome. NK cells have been recently included in a wider innate lymphoid cell (ILC) family, and circulating cells are just the tip of an iceberg formed by a conspicuous and heterogeneous lymphoid population colonizing both, lymphoid and non lymphoid tissues (1–3). Moreover, cytometry by time-of-flight (CyTOF) highlighted the existence in peripheral blood (PB) of a single individual of at least 30,000 different NK cell phenotypes (4). These findings consolidate the concept that observed phenotypic and functional NK cell status actually represents a single crystalized picture of a very dynamic process. Nevertheless, in healthy individuals, two main circulating PB NK cell populations have been extensively studied, CD56^bright^ and CD56^dim^ NK cells, which represent sequential stages of maturation and show a dichotomy in phenotypic and functional properties (5). These include the expression of MHC class I-specific inhibitory Killer Ig-like Receptors (KIRs), restricted to CD56^dim^ NK cells that represent the majority of cells circulating in blood. KIRs are involved in NK cell “education,” a phenomenon that provides the basis of self-tolerance and generates “armed” cells, i.e., NK cells fully responsive to the engagement of activating receptors (i.e., NCR, NKG2D, and DNAM-1) (6, 7). CD56^dim^ NK cells also express high levels of CD16, thus exerting strong antibody-dependent cellular cytotoxicity (ADCC). Moreover, they efficiently respond to cytokines stimulation and are characterized by a chemokine receptor repertoire giving them the potential to colonize lymphoid and non-lymphoid tissues in response to a proper chemokine milieu.

The composition of the milieu can greatly vary in perturbed tissues. This justifies the prevalence in some tumors of immature, poor cytolytic CD56^bright^ NK cells that are undetectable in matched healthy tissues (8). Tumor parenchyma, as well as the immune cells participating to the inflammatory processes, may change the microenvironment providing NK cells with a plethora of stimuli. These include membrane-bound or soluble molecules such as chemokines or cytokines (TGF-β, IL-12, IL-18), which either promote or dampen innate and adaptive immune responses. Cytokines, in addition to shape the functional activity of NK cells, modify their chemokine receptor repertoire altering their native migratory potential (9–13) and at the same time provide signals essential to generate, expand and recall memory NK cell populations (14). Interestingly, recent data showed that non-hematopoietic organs such as liver can be colonized by peculiar tissue resident NK cell populations that belong to the memory NK cell reservoir able to mediate "recall” responses (15).

Here, we will recapitulate studies that analyzed the main mechanisms regulating NK cell trafficking in lymphoid and non-lymphoid tissue under either steady state or “perturbed” conditions, including tumors, inflammation and pregnancy.

## Defining dynamics of NK cells in healthy tissues

NK cells are not exclusively found in PB but populate different tissues and organs. The traditional view of NK cells as “armed” effector cells, which patrol human body through blood ready to extravasate to the site of injury, has been partially revisited and a growing number of studies show that NK cells might also stably reside in most peripheral tissues, under steady-state conditions.

**Figure 1:**
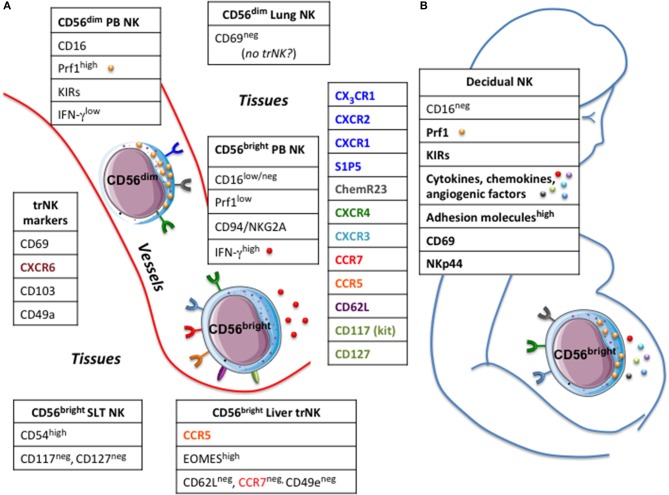
Circulating and tissues resident NK cells. **(A)** In human two main NK cell subsets, CD56bright and CD56dim, can be detected in peripheral blood (PB NK) having a different repertoire of chemokine receptors. Tissue resident (tr) NK cells share the expression of certain markers but express molecules typical of the hosting tissue.**(B)** large numbers of NK cells populate the decidua, particularly in the first trimester of pregnancy. Decidual NK cells have unique phenotypic and functional characteristics, which contribute to support nutrition of the fetus, ensure maternal-fetal tolerance and control viral infections. Prf1, perforin; KIRs, killer cell immunoglobulin-like receptors; SLT, secondary lymphoid tissues.

Until recently, the task of depicting NK cell distribution in human compartments has suffered from several methodological shortcomings. Earlier analyses often relied on the use for NK cells detection of markers poorly specific and/or unable to distinguish the two main NK cell subsets, i.e., CD56^bright^ CD16^low/neg^ Perf^low^ and CD56^dim^ CD16^pos^ Perf^high^. The advent of new OMICS technologies, and the possibility to perform single-cell analyses have expanded our understanding on the distribution of NK cells across human body. Indeed, in the recent years, our knowledge about NK cell diversity has further increased with the identification of NK cell subsets specifically populating various peripheral solid organs, such as lung, liver, lymphoid tissues, and uterus. These findings have challenged the classical view of NK cells as a lineage comprising a relatively homogeneous population of cells with similar functions and longevity. Nonetheless, at variance with B and T cells, we know little about recirculation and trafficking of NK cells across peripheral tissues. Although NK cells express an ample array of chemotactic receptors, the role of the different chemokines in guiding *in vivo* the distribution of NK cells through the body compartments still remains unclear. The distribution of NK cells seems to be subset-specific in mouse, as different NK cell subsets showed organ-specific localizations (16). Conversely, this issue has been poorly investigated in the human system. As the two major PB-NK cell subsets display a chemokine receptors pattern that only partially overlaps, they may have a peculiar tissue-specific compartmentalization (Figure 1). PB-CD56^bright^ NK cells are uniquely characterized by the expression of CCR7, CXCR3, and L-selectin (CD62L), which justify their abundance in secondary lymphoid tissues (SLTs). Conversely, PB-CD56^dim^ NK cells, despite sharing the CXCR4 receptor with CD56^bright^ NK cells, are equipped with receptors specific for inflammatory chemokines, such as CXCR1, CXCR2, CX_3_CR1 (8, 16, 17). Additionally, CD56^dim^ NK cells can migrate in response to factors that do not belong to the chemokine superfamily. These include the proinflammatory protein chemerin and the sphingosine 1-phosphate (S1P) molecule that affect trafficking of NK cells during inflammation or steady-state conditions, respectively (18, 19). Based on the different expression of chemotactic receptors, the tissue distribution of human NK cell subsets observed under steady-state conditions is dependent on the expression of local tissue-specific environmental signals. In order to shed light on the mechanisms lying behind the migratory properties of PB NK cells, a wide array of samples derived from different body compartments was analyzed to investigate the presence and distribution of functionally different NK cell subsets (8). The study showed that the relative distribution of CD56^bright^ and CD56^dim^ NK subsets in the various human districts does not parallel that in PB. CD56^dim^ NK cells represent the major NK cell subset in bone marrow (BM), lung, spleen, subcutaneous adipose tissue and breast tissue, whereas CD56^bright^ NK cells abundantly outnumber cytotoxic NK cells in gastric and intestinal mucosa associated lymphoid tissues (MALTs), liver, uterus, visceral adipose tissue, adrenal gland, and kidney (8, 20, 21). Importantly, the relative distribution of the two main NK cell subsets matched with the specific patterns of chemotactic factors expressed in the tissues (8).

A main question arising from the detection of NK cells in many organs is whether NK cells stably reside in those tissues or could eventually exit and recirculate. Studying the dynamics of NK cells under steady-state conditions is limited by the difficulty of having access to samples from human body districts. On this regard, useful hints may be derived from studies in which human subjects have been treated with monoclonal antibodies directed against molecules pivotal in lymphocytes migration, such as integrins. This is the case of natalizumab, a humanized monoclonal antibody directed against the α4-chain of VLA-4 (α4β1) and α4β7 integrins, widely expressed on many different lymphocyte populations including T cells, B cells, and NK cells as well as on a majority of monocytes and macrophages. Interestingly, it has been reported that 1-year treatment with natalizumab in multiple sclerosis patients resulted in a pronounced accumulation (almost 2-fold increase compared to baseline levels) of NK cells in PB (22), which then gradually decreased upon treatment interruption (23). These data are in evident agreement with a dynamic passage of circulating NK cells across the endothelial barriers for patrolling peripheral tissues, although it remains to be determined whether it might occur also in steady-state or just under inflammatory conditions.

In addition to extravasation from PB to solid tissues, NK cells may eventually egress from peripheral tissues and trafficking to SLT. This re-circulation has been suggested by the direct investigation of afferent lymph draining from normal skin (24) and analysis of cellular content in seroma fluid upon axillary lymph nodes (LN) dissection, which represents an accumulation of *bona fide* afferent lymph (25, 26). Interestingly, most seroma NK cells expressed high level of CCR7 and CD62L, as well as CXCR4, CXCR3, a chemokine receptor repertoire identifying lymphocyte populations migrating toward SLTs. These data indicate that high endothelial venules (HEVs) might not represent the only route for NK cell entrance in SLTs. Conversely, very little information is available regarding the egress of NK cells from SLTs. It has been described in the murine model that changes in responsiveness of sphingosine-1 phosphate receptor 5 (S1P5) to its ligand (S1P) play a key role in allowing NK cell egress via lymphatics (27). However, whether this mechanism might also be effective in human has not yet been confirmed. Notably, NK cells have been detected in efferent lymph fluid and NK cells exiting from LN have a phenotype slightly different from that of NK cells found within SLTs. In particular, a portion of NK cells express significant amounts of KIR and CD16, implying that CD56^bright^ NK cells might acquire these molecules in the LN during inflammation and then egress through the efferent lymph for recirculating in PB (28).

All these previous studies have so far depicted the distribution of the two main “conventional” human NK cell subsets across the human body (8, 29). Recently, this issue reached a higher level of complexity because of data showing that various body districts harbor “unconventional” subsets of NK cells that apparently do not recirculate in the blood or lymphatics and adopt a unique phenotype that is distinct from that of circulating NK cells. Tissue residency has been described for NK cells as well as for other “helper” innate lymphoid cells (ILCs), T cell subsets (memory CD8, CD4 and Treg cells) and “innate-like” T cell types, including subpopulations of γ/δ T cells and natural killer T (NKT) cells (30). Tissutal NK cells, similarly to other lymphocytes residing in tissues, may display markers such as CD69, CD103 (also known as αE integrin) and CD49a (also known as α1 integrin), which are functionally involved in retaining them in tissues and, hence, can be useful for the identification and isolation of tissue-resident (tr) NK cells (Figure 1). As discussed earlier, at least three-quarters of NK cells in non-reactive lymph nodes display a CD56^bright^ Perf^low^ phenotype (20, 31). This accumulation is compatible with the pattern of adhesion molecules (CD62L) and chemokine receptors (CCR7) expressed on circulating PB-CD56^bright^ NK cells but not PB-CD56^dim^ NK cells. From recent data, it is possible to speculate that a fraction of NK cells reaching the LN could be retained within the structure as trNK cells. Supporting this hypothesis is the presence of a distinct subset of NK cells in human SLTs characterized by co-expression of CD69 and CXCR6, high expression of CD54 (ICAM-1) but lacking CD117 (c-kit) and CD127, the latter specifically expressed by CD56^bright^ NK cells (32). Because of the high level of CD54, these SLT-NK cells are also reminiscent of CD56^bright^ NKG2A^pos^ CD94^pos^ CD54^pos^ CD62L^neg^ NK cells that accumulate in tonsils of EBV carriers, which produce high amount of IFNγ, show very low plasticity even after prolonged cytokine stimulation, and are able to potently restrict EBV-induced transformation of B cells (33).

Among solid tissues, liver is abundantly populated by NK cells, where they represent up to 30–40% of all the lymphocytes populating this organ (34). At steady-state, NK cells are preferentially located in the hepatic sinusoids, often adhering to the endothelial cells (35). Similar proportion of CD56^dim^ and CD56^bright^ NK cell populations have been reported to populate this organ (36), but only CD56^bright^ has been described to own features of trNK cells. Indeed, liver CD56^bright^ NK cells are characterized by higher level of EOMES transcription factor, expression of CXCR6 and CD69 as well as CCR5 but absence of CD62L and CCR7 (37). Interestingly, the expression of CD49e (also known as α5 integrin or VLA-5 α chain) has been recently identified has a reliable marker able to distinguish conventional “circulating” NK cells from *bona fide* liver-NK cells, which are otherwise negative for this marker (38). Many reports have suggested the importance of CCR5 and CXCR6 in their localization and retention within liver parenchyma, since their cognate ligands (CCL3, CCL5, and CXCL16) are constitutively expressed by various parenchymal and non-parenchymal cells in the liver, including cholangiocytes, sinusoidal endothelial cells, hepatocytes and Kupffer cells (34). Investigation of human liver transplants has indicated that EOMES^high^ trNK cells can persist *in situ* for very long periods (up to 13 years in one human study), further supporting the idea that subsets of NK cells may stably reside within liver tissues. At the same time, circulating CD56^bright^ EOMES^low^ cells may be recruited to the liver and have the potential to become CD56^bright^ EOMES^high^ NK cells (39).

An exception to the aforementioned tissues is represented by lungs since: (i) the majority (~80%) of NK cells populating these organs belongs to the CD56^dim^ Perf^high^ subset (40); (ii) only a limited fraction of Lung-NK cells is characterized by expression of markers consistent with tissue-residency (i.e., CD69). Interestingly, this fraction is mainly composed of CD56^bright^ CD16^neg^ and only a small proportion of CD56^dim^ CD16^bright^ NK cells (41), thus suggesting that “genuine” lung-resident NK cells may share some commonalities with CD56^bright^ trNK cells found in the uterus, liver, and lymphoid tissues (37). Lung-NK cells were detected in the parenchyma only, and were not found outside of the parenchyma, (i.e., blood vessels or bronchi) (8, 41). Therefore, overall, these data support a model in which human lungs mainly contain highly differentiated NK cells recirculating between lung and blood, rather than a stable pool of tissue-resident NK cells (41). Consistent with this hypothesis, using a parabiotic mouse model, it has been recently shown that parabiont-derived donor NK cells are able to rapidly replenish the majority of NK cells in the lungs of recipient mouse (42).

Development of tissue-resident lymphocytes seems to involve a transcription program inducing the expression of genes involved in tissue-retention while inhibiting that of genes important for tissue egress and trafficking. In mice, it was recently described that the transcription factor Hobit (homolog of Blimp-1 in T cells or ZNF683), a zinc finger protein, acts in concert with Blimp-1 (B lymphocytes-induced maturation protein) to serve as a master regulator of tissue-residency for lymphocytes. Thus, Hobit and Blimp-1 mediate a common transcriptional program that is shared among tr memory (Trm) T cells, NKT, trNK cells, and helper-like ILCs. Together with Blimp-1, Hobit sustain unresponsiveness to signals for SLT re-circulation from peripheral tissues by suppressing expression of *S1pr1* (which encodes S1P1), *Sell* (which encodes CD62L) and *Ccr7* (which encodes CCR7) (30). The role of Hobit in human Trm cells is less clear. Recent reports have shown peculiar results with regard to the expression of Hobit/ZNF683 in the two major human PB-NK cell subsets. Indeed, Hobit has been detected at high levels in circulating CD56^dim^NK cells (despite this transcription factor is almost absent in circulating NK cells in mice) while only poorly expressed by PB-CD56^bright^ NK cells (43, 44).

However, it has been found that a strong Hobit/ZNF683 expression identifies a subset of intrahepatic CD56^bright^ NK cells in human liver, which additionally express a distinct set of adhesion molecules (CD69, CD49a) and chemokine receptors (CXCR6) consistent with tissue residency (44). These data may suggest that Hobit expression in humans may instruct unique migratory properties in the two distinct circulating NK cell subsets. Whilst low expression of Hobit in circulating CD56^bright^ NK cells could maintain high levels of CCR7 and CD62L necessary for SLT entry, high level of Hobit in CD56^dim^ and CD56^bright^ trNK cells might down-regulate these markers on their surface, thus limiting their recirculation to SLT and tissue egress, respectively.

## NK cells in pregnancy

Pregnancy is a quite peculiar situation, in which an immunocompetent individual (the mother) is in contact for a long period of time with a genetically different immunodeficient individual (the fetus), and is characterized by a deep modification of mother's tissues. During the first trimester of pregnancy, extravillous trophoblast cells (EVT) from the fetus invade the maternal decidua penetrating through the basement membrane of the uterus epithelium with remodeling of the maternal spiral arteries. These changes ensure adequate nutrition of the fetus and are supported by immune cells present at the maternal-fetal interface (45). In normal pregnancy many different mechanisms exist to ensure tolerance of the semi-allogeneic fetus by the maternal immune defense, thus preventing fetus rejection and allowing the reproductive success.

The decidua is populated by a large variety of leukocytes, which represent approximately 30–40% of decidual cells. The most represented leukocyte populations are NK cells, CD14^pos^ myelomonocytic cells and T lymphocytes (46). Decidual NK cells (dNK) represent 50–90% of total decidual lymphoid cells in the first trimester of pregnancy (47) (Figure 1). The number dwindles by the end of second trimester, and returns to basal levels at the end of pregnancy. NK cells have also been identified in non-pregnant endometrium (eNK) and their number changes throughout the menstrual cycle, reaching the maximal level in the post-ovulatory phase of the cycle (48). Most uterine NK cells do not express CD16 and show high levels of CD56. The dNK cells have been shown to exhibit unique phenotypic and functional properties. Indeed, relevant differences exist in the gene expression of the NK cell subsets present in peripheral blood and early pregnancy decidual tissues. CD9 tetraspanin, galectin, α-1 integrin and other adhesion molecules are overexpressed in dNK (49). Unlike resting PB NK cells, dNK cells express the CD69 marker and a large percentage express the NKp44 activating receptor. The expression levels of activating receptors/co-receptors (NKp46, NKp30, DNAM-1, NKG2D, and 2B4) are similar in dNK and PB NK cells and, regarding to inhibitory MHC class I-specific receptors, the dNK cells have been shown to express Killer Immunoglobulin receptor (KIRs), CD94/NKG2A and LILRB1 (also known as ILT2, LIR1, and CD85j). Interestingly, the KIR repertoire of dNK cells is skewed toward recognition of HLA-C, the only classical MHC Class I molecule expressed by trophoblast cells (46, 50). Although expressing both perforin and granzymes dNK cells are poorly cytotoxic, a characteristic that has been linked to the block in the polarization of cytolytic granules to the immunological synapse (51). Importantly, cytokines, such as IL-15 can restore the dNK cell cytotoxic function, a phenomenon that is crucial in normal pregnancy to control viral infection (52).

Various studies have shown peculiar functional capabilities of dNK cells. Indeed, they release a wide panel of cytokines, chemokines, and angiogenic factors that are involved in the development of placenta, tissue remodeling, trophoblast invasion and neoangiogenesis (48). Several studies analyzed the chemokine repertoire in endometrium and decidual tissues of women undergoing elective pregnancy termination, studying its involvement in NK recruitment. CXCL9 (Mig), CXCL10 (IP10), CXCL12 (SDF-1), CCL3 (MIP-1α) e CCL4 (MIP-1β) are constitutively expressed in the endometrium. First-trimester human trophoblast expressed and released chemokines able to exert their activity on NK cells, including CXCL12 and CCL3 (53). In line with these results studies have shown that chemokines produced by endometrial or trophoblast cells induce the peripheral blood NK cell chemotactic response. Decidual endothelial and stromal cells express CCL2 (MCP-1), CXCL8 (IL-8), CXCL10, CX_3_CL1 (fractalkine), and CXCL12 while only stromal cells express detectable levels of CCL5 (Rantes) and CCL4. Noteworthy, CXCL10, CXCL12 and CX_3_CL1 induce the migration of PB NK cell across primary cultures of decidual endothelial and stromal cells (54). Furthermore it has been shown that also chemerin is expressed in the uterus by EVT and stromal cells but not by decidual endothelial cells (DEC) (55–58). The treatment of DECs and stromal cells with progesterone enhanced CXCL10, CX_3_CL1, and CCL2 but not CXCL12 levels, while estrogen treatment of stromal cells resulted in up-regulation of CXCL10 and CX_3_CL1 (54–57). Moreover, the treatment of stromal cell primary cultures from pregnant, fertile non-pregnant, or menopausal women with progesterone and estrogen resulted in a significant up-regulation of chemerin secretion.

Although it is unclear how and when the various chemokines participate in the recruitment of dNK cells, it has been shown that dNK cells express high levels of CXCR3, low level of CXCR4 and very low levels of CXCR1, CXCR2, CX_3_CR1 or CCR1, 2, 3, 5, 6, and 7. In this regard, CXCR3 and CXCR4 are involved in migration of decidual NK cells to CXCL9, CXCL10 and CXCL12 respectively (59). Moreover dNK cells migrate through stromal cells in response to CXCL10 and CXCL12 but not to CX_3_CR1 (54). Interestingly, dNK cells from pregnant women express chemerin receptor (ChemR23 or CMKLR1) that induces their migration through stromal cells in response to chemerin. The different chemokine receptor profile between dNK and PB NK cells suggests that the phenotypic features of leukocytes recruited from peripheral blood during pregnancy can be influenced by the decidual microenvironment. In this regard, evidence indicates that the pregnant uterus is a good source of cytokines acting on NK cells including IL-15 (60). Interestingly *in vitro* culturing of PB NK cell with IL-2 or IL-15 induced a down-regulation of ChemR23 (18). In line with these observations studies have shown that co-culture of PB NK cells with stromal cells results in a chemokine receptor profile similar to that of decidual NK cells (54).

Nevertheless, it is noteworthy that the precise origin of dNK cells is not yet clear. It is possible to speculate that a pool of dNK cells may originate from PB NK cells recruited in decidua at early stages of pregnancy. On the other hand, studies suggest that they could also originate from *in situ* progenitor cells that, in response to uterine stromal environment, differentiate into CD56^bright^ CD16^neg^ NK cells (61).

## NK cells in tumor tissues

**Figure 2:**
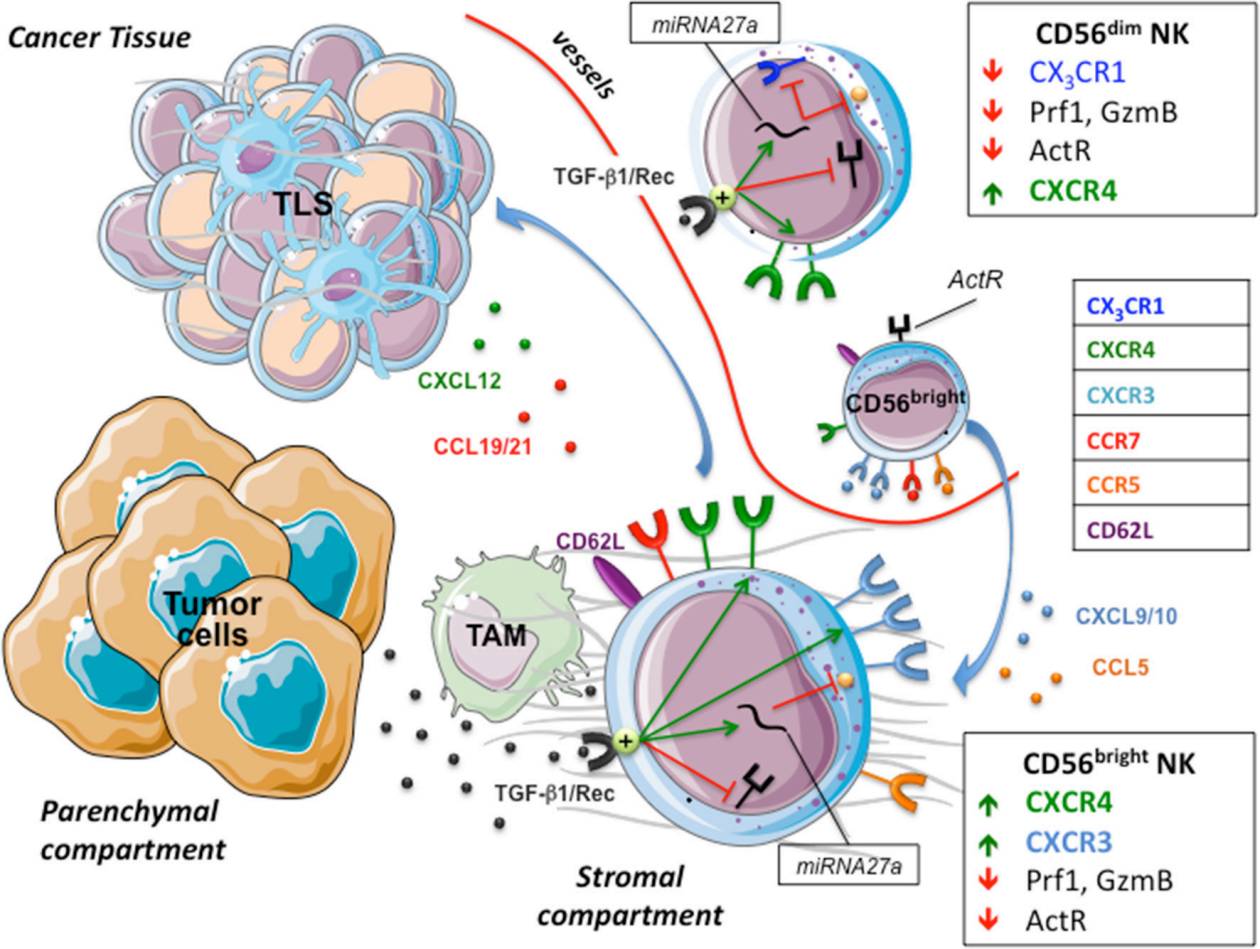
NK cells in tumor microenvironment. The tumor chemokine milieu presents a reduced expression of chemokines attracting CD56dim NK cells, and an increased expression of CXCL9/10, CCL5, and CXCL19/21 that drives the migration of CD56bright NK cells toward the stromal compartment and tertiary lymphoid tissue (TLS). Tumor cells and tumor-associated macrophages (TAM) release/activate TGF-β1 that decreases the capability of NK cells to recognize and kill targets and modify their chemokine receptor repertoire, hampering the recruitment of CD56dim NK cells and favoring that of poor cytolytic CD56bright. ActR, Activating receptors; GzmB, Granzyme B.

A consolidated view considers NK cells as the more effective lymphocyte subset involved in immune surveillance of hematological malignancies, initial stages of solid tumors and blood spreading metastatic cells (62–64). Conversely, NK cells appear to be poorly efficient in controlling advanced, consolidated tumors due to different reasons, which comprise the plethora of immune suppressive factors characterizing the tumor microenvironment (63, 65). These include the expression by cancer cells of MHC class I molecules and immune checkpoint-ligands such as PD-Ls and B7-H3 (63, 66), the lack of expression or the release of soluble forms of ligands of activating receptors, and the presence of soluble immunomodulators, the prototypic one being represented by TGF-β1 (63, 65). Additional aspects impacting on the NK-mediated tumor immune surveillance are the low frequency and/or the quality of NK cells attracted in tumor tissues (Figure 2). Indeed, highly cytolytic CD56^dim^ CD16^pos^ NK cells are rare and immature CD56^bright^ CD16^low/neg^ NK cells with low perforin content represent the majority of tumor-associated NK cells. Although some authors suggested the possibility of an *in situ* expansion of CD56^bright^ NK cells (67), a shared hypothesis considers as primum movens the type of chemokines/receptors interactions occurring in the tumor microenvironment.

The tumor orchestrates escape strategies and creates a chemokine milieu consisting of reduced expression of CXCL2, CX_3_CL1, CXCL1, and CXCL8, attracting CD56^dim^ NK cells, and increased expression of CXCL9, CXCL10, CXCL19, and CCL5 that drives migration of CD56^bright^ NK cells. The dichotomy between high (CD56^dim^) and low (CD56^bright^) cytolytic NK cells has been widely studied and data show that pro-inflammatory cytokines can increase the killing properties of CD56^bright^ NK cells (68). However, this cytokine-mediated rescue mechanism might be deeply affected by TGF-β (69), which is highly represented in tumor tissues. This is because the tumor environment is rich in both TGFβ-1 producing cells and in factors that induce TGFβ activation, such as acidic pH, reactive oxygen species, proteases and specific members of integrin family (70). Active TGF-β1 decreases the expression of activating NK receptors and, by up-regulating mir27a-5p, of perforin 1 (Prf1) and granzyme B (GzmB), thus hampering NK cell cytotoxicity. Moreover, TGF-β1 might dampen CD56^dim^ recruitment and favor that of CD56^bright^ by modifying their respective chemokine receptor repertoires (13). In particular, TGF-β1 increases the expression of CXCR3 and CXCR4 in CD56^brigh^ and CD56dim NK cells, whereas, via mir27a-5p, down-regulates CX_3_CR1 expression in CD56^dim^ cells (13, 71). CX_3_CR1, whose cognate ligand is represented by CX_3_CL1 (also known as fractalkine), is selectively expressed by CD56^dim^ NK cells and together with CXCR4 has been demonstrated to regulate NK cell-egress from bone marrow and NK cell extravasation (72). Interestingly, in agreement with this ability of tumors in inducing a regulatory milieu, an unusual low expression of CX_3_CR1 has been reported in CD56^dim^ NK cell population of tumor-infiltrated bone marrow and peripheral blood of Neuroblastoma (NB) patients (13). Although a more detailed analysis should be performed to deepen whether this unusual chemokine receptor repertoire actually defines a peculiar CD56^dim^ population (73) mirroring the “broad spectrum of human Natural Killer Cell Diversity” (2), it is conceivable that CX_3_CR1^low^ CD56^dim^ cells show defective migration toward tumor (or inflamed tissues). Conversely, the recruitment of CD56^bright^ NK cells in a CXCL9 and CXCL10 rich milieu might be favored by their constitutive expression of high levels of CXCR3 and CXCR4, which further increase under the influence of TGFβ-1 (8, 16, 17). Along this line, CD56^bright^ CD16^low^ represented the predominant NK cell population in the ascitic fluids of ovarian cancer patients (74). The concomitant up-regulation of CXCR3 and CXCR4 by TGF-β1 represents an interesting event if considering that these receptors are subject to cross regulation. Indeed, chemokine receptors' function can be modulated by desensitization, which is a physiological process that prevents overstimulation due to prolonged agonist exposure by signal attenuation or termination (27). Desensitization of a receptor can be dependent on its ligand (homologous desensitization) or by other ligands present in a complex chemokine gradient, a cross-desensitization called heterologous desensitization. In this context it has been shown that pre-stimulation of NK cells with CXCL9 inhibited NK cell migration not only to CXCR3 ligands but also to CXCL12, thus indicating that triggering of CXCR3 can promote both homologous and heterologous (CXCR4) desensitization (75).

In solid tumors a “fast track entrance” for CD56^bright^ CX_3_CR1^neg^ CXCR3^high^ CXCR4^high^ NK cells might be the ectopic, neo-generated High Endothelia Venules (HEV) that contribute to the architecture of Tertiary Lymphoid Structures (TLS) (76, 77). These transient, un-capsulated lymphoid aggregates resembling Secondary Lymphoid Organs (SLO) have been detected in peri- or intra-tumor sites as well as in other chronic inflamed tissues. TLS share with SLO the presence of distinct T and B cell compartments, reactive Germinal Center (GC), Follicular Dendritic Cells (FDC), fibroblastic reticular cells (FRC) and lymphatic vessels, as well as HEV whose lining endothelial cells express highly specific addressin molecules, collectively termed peripheral node addressins (PNAd) (76). These are known to dictate adhesion and consequent extravasation of immune cells, including NK cells, within paracortical region of lymph nodes, an event that might occur also at TLS levels. In different tumors including lung, breast or gastrointestinal stromal tumors (GIST) (78), tumor-associated TLS might contribute to the preferential recruitment of CD56^bright^ NK cells that constitutively express the homing receptor CD62L and high levels of CCR7 specific for the lymph node chemoattractants CCL19 and CCL21. For example, in TLS associated to human lung cancer intra-tumoral PNAd^+^ HEV exclusively co-localized with CD62L^+^ lymphocytes (76). Notably, while TGF-β negatively impacts on CD56^dim^ NK cells recruitment in perturbed tumor tissues, upregulation of CCR7 may promote their migration to SLO and TLS. Accordingly, enrichment in CD56^dim^ CCR7^+^ KIR^+^ CD57^+^ highly cytotoxic NK cells has been documented in tumor-infiltrated lymph nodes of melanoma patients (79). Several mechanisms involved in the acquisition of CCR7 by CD56^dim^ NK cells have been identified that include the crucial role of IL-18, highlighted by Mailliard et al. (80), and the possible uptake of CCR7 from surrounding cells by trogocytosis (81). Soluble IL-18 is produced by stimulated antigen presenting cells, in particular by macrophages that, as M2-polarized cells, might represent the most abundant immune population in the tumor microenvironment (82). Interestingly, a variable subset (30–40%) of unpolarized (M0) and M2 macrophages and most tumors associated macrophages (TAM) express a membrane form of IL-18 (mIL-18) (74, 83, 84). Upon TLR stimulation, macrophages polarize toward M1 and loose mIL-18, an event paralleled by the release of small amounts of soluble IL-18 (sIL-18) that, acting in close proximity, induces the expression of CCR7 in CD56^dim^ NK cells (83). It is of note that, since M1 polarizing macrophages also acquire CCR7 expression (83), a contribution of trogocytosis-mediated uptake cannot be ruled out. Although mechanisms responsible for IL-18 membrane retention and release have to be clarified, this cytokine shows many predictable cleavage sites for extracellular proteases such as Matrix metallopeptidase (MMP) −2 and −9, which characterize the secretory profile of parenchymal tumor cells and TAM. Thus, also in the absence of pathogen-derived stimuli, the action of MMPs (or other still unknown mechanisms), may allow IL-18 shedding from TAM and the induction of CCR7 expression in CD56^dim^ tumor-associated NK cells (TA-NK), thus promoting their migration to SLO and TLS.

In solid tumors CCR7 acquisition by NK cells may depend on close cell-to-cell contacts with macrophages or dendritic cells, whereas it is less plausible that tumor cells could play a relevant role. Indeed, TA-NK cells were found to be predominantly located in the stromal compartment, whereas they were rare/absent in the parenchyma in direct contact with tumor cells (40, 85). Regarding the compartmentalization of TA-NK cells, in an adenocarcinoma colon model, stromal-infiltrating NK cells had morphology compatible with actively migrating cells, and in some instances migrating NK cells co-localized with degraded matrix (85). In the same model, most of the NK-poor tumor nodules were surrounded by a capsule-like structure with collagen IV and laminin, two major components of the basement membrane. On the contrary, tumor nodules lacking these containment structures were more infiltrated by NK cells. These observations, together with data showing that poor NK cells infiltration have been equally detected in both chemokines-rich and -poor tumors, strongly indicate stromal barriers as a hindrance impacting on possible NK-to-tumor cell contacts. Along this line, during imatinib mesylate therapy in GIST patients, the frequency of NK cells did not change in fibrous trabeculae, whereas significantly increased in the core of both localized or metastatic tumors, an observation that correlated with a better prognosis (78). Interestingly, a recent study analyzing the off-target effect of imatinib mesylate on immune cells showed that this drug causes a significant up-regulation of CXCR4 in both T and NK cells (86). Accordingly, NK cells *ex-vivo* isolated from peripheral blood of chronic myeloid leukemia patients receiving imatinib mesylate showed levels of CXCR4 significantly higher than those detected in healthy individuals (86). A study by Goda S. and colleagues (87) may in part explain how increased CXCR4 surface levels can facilitate NK cells to cross the bridge connecting the stroma and the tumor parenchyma compartments. In particular, they showed that human CD56^dim^ CD16^pos^ NK cell invasion into type I collagen is enhanced by CXCL12, the CXCR4 ligand, in a matrix metalloproteinases (MMP)-dependent manner. Notably, CXCL12 has been shown also to promote the production in monocytes (88) and megakariocytes (89) of MMP-9, which has protease activity on collagen IV. With this assumption, it is conceivable that therapies strengthening the CXCR4/CXCL12 axis could potentiate extracellular matrix degradation favoring NK (and T) cells migration toward tumor cells.

In light of these considerations, data on the NK cell phenotype and density in tumor sites cannot be considered “*per se*” a favorable prognostic factor and should be more and more integrated with data on NK cell localization with respect to stroma, parenchyma tumor cells and with the analysis of the whole immune landscape. For instance, high NK cell infiltration has been associated with improved survival in metastastic renal cell carcinoma but not in colorectal carcinoma (90). Contradictory results may depend on the method used to unequivocally identify NK cells, which still represents a major challenge as NKp46, the more reliable marker, is also expressed by other subsets of ILCs (91). Opposite clinical impact of NK cell infiltration in solid tumors might also depend on the targeted tissue, the tumor phase and the ratio between NK and tumor cell numbers. It has been demonstrated that NK cells can edit tumor cells modifying their immunogenicity. In particular, in NK and melanoma cell co-cultures performed at low effector/target ratios, which reflect the level of NK cell infiltrates observed at the tumor site, an initial tumor cell lysis is followed by an equilibrium phase characterized by decreased susceptibility to killing due to up-regulation of both classical and non-classical MHC class I molecules on melanoma cells. This effect is mediated by IFN-γ released by NK cells activated upon melanoma cell recognition. Importantly IFN-γ and TNF-α are also potent inducers of the expression of the immune checkpoint ligands PD-L1 and PD-L2 in macrophages/dendritic cells, tumor cells and tumor-associated endothelial cells (92, 93). Moreover, TNF-α is known to promote Epithelial-Mesenchymal Transition (EMT), a process leading epithelial tumors to acquire a less differentiated, pro-metastatic phenotype. Along this line, in lung cancer, a recent report showed an important correlation between PD-L1 expression and EMT score (93, 94). Thus, low number of NK cells contacting tumor cells might have more undesirable than beneficial effects, being unable to efficiently eliminate tumor cells while causing a gradual accumulation of cytokines that exert a paradoxical tumor promoting effect by modifying the immunogenicity of tumor cells.

Whatever the case, when designing NK cell-based immunotherapeutic approaches for cancer patients, we should take into account the relevance of the molecular mechanisms regulating NK cell migration into tumors. For instance, a recent and promising approach is represented by the infusion of NK cells engineered to express chimeric antigen receptor (CAR) specific for tumor-associated antigens (65, 95, 96). The efficacy of adoptively transferred CAR-NK might be deeply limited by their inability to cross stromal barriers and to adhere to parenchymal tumor cells, as recently suggested for T cells by Caruana and colleagues (97). It was pointed out that *in vitro* manipulation aimed to the CAR engineering of T cells leads to silencing of heparanase (HPSE), an endoglycosidase that cleave heparan sulfate proteoglycans of ECM, thus reducing the invasive potential of CAR-T cells in solid tumors. Thus, cell-based therapy may also include strategies to favor migration of effector cells through stromal compartment and tumor parenchyma, a phenomenon unlikely to occur, particularly in advanced solid tumors.

## NK cells in inflamed tissues

The perturbation mediated by pathogens in peripheral tissues results in the early activation of resident or recruited cells of the innate immunity with a consequent boost of chemotactic factors, which attract different immune cells including peripheral blood mature conventional CD56^dim^ NK cells. These cells mainly differentiate in the bone marrow and express CXCR1, CXCR2, chemR23, S1P5, CXCR4, and CX_3_CR1 (16, 17, 26). This chemokine receptor repertoire drives NK cells to inflamed tissues. Importantly, the relative expression of CXCR4 and S1P5 in developing mouse NK cells has been described to regulate bone marrow egress into circulation (98). Moreover, in bone marrow, prevalent CX_3_CR1 expression by KLRG1^+^ NK cells located in sinusoids suggested its crucial role for NK cell entry into the vascular compartment (99).

**Figure 3:**
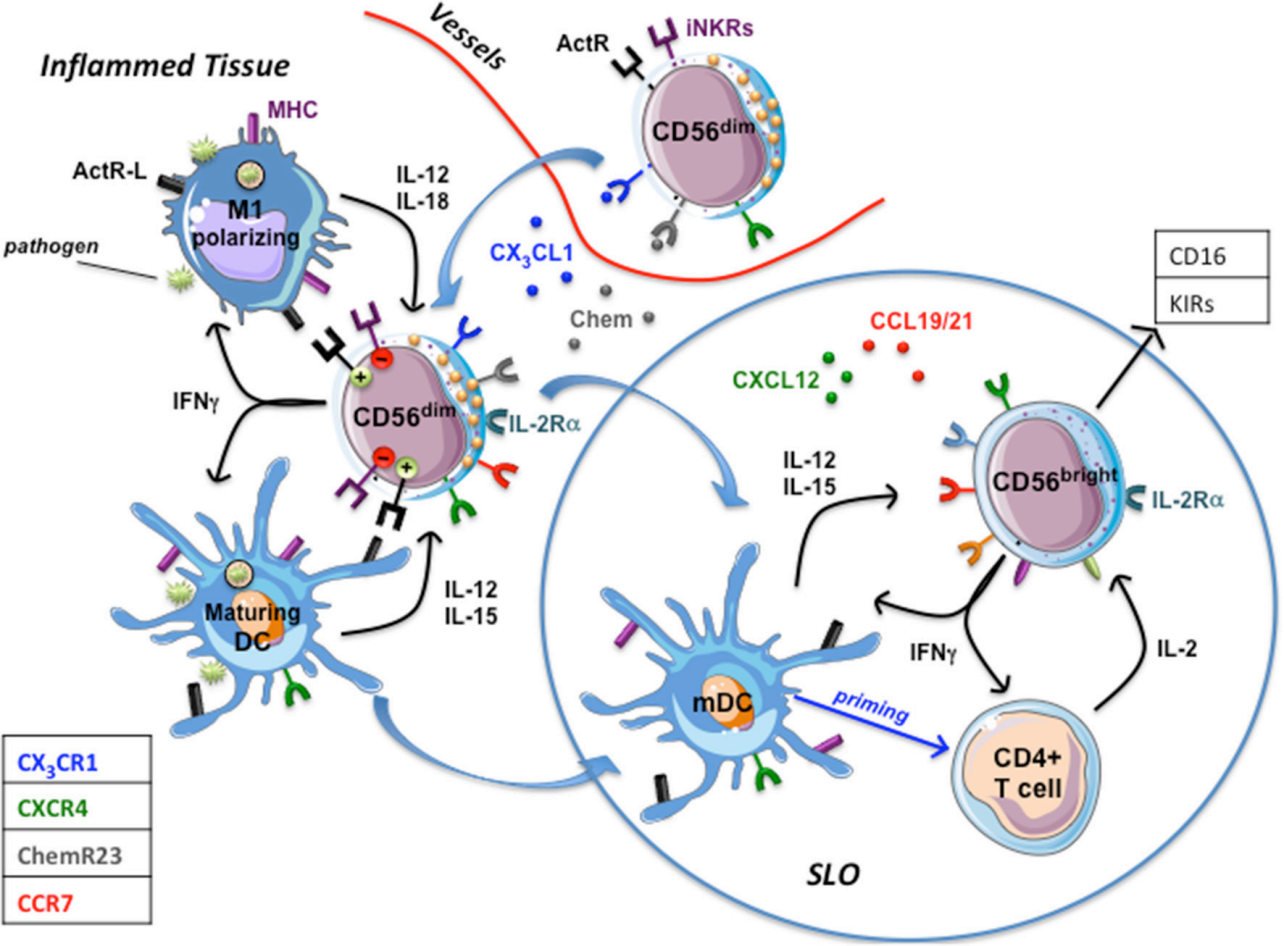
NK cells in inflamed tissues. Inflamed tissue produce chemokines (Chemerin, CX3CL1) that drive the migration of circulating CD56dim prf1high NK cells. Once in tissues they interact with DC and macrophages that, upon pathogen recognition, have begun to mature (mDC) or polarize toward a proinflammatory functional phenotype (M1). Maturing DC and M1-polarizing macrophages release immunostimulatory cytokines that induce NK cells to produce large amounts of IFN-γ (which potentiate phagocytes' functions) and to express IL-2Rα and CCR7, which drive their migration into secondary lymphoid organs (SLO). mDC migrated to SLO and DC-primed T cells producing IL-2 stimulate CD56dim NK cells and CD56bright NK cells that acquire a CD16^pos^ KIR^pos^ phenotype. ActR-L, Activating receptors ligands; iNKRs, MHC class I specific inhibitory receptors (KIRs, CD94/NKG2A); Chem, chemerin.

The presence of NK cells in healthy and inflamed peripheral tissues (18, 26, 29) has been well documented and different studies demonstrated the existence of a crucial crosstalk between CD56^dim^ NK cells and DC or macrophages (Figure 3). NK/DC interactions resulted in a bidirectional activation leading to killing of immature DC (iDC) by autologous NKG2A^+^ KIR^neg^ NK cells. NK-mediated DC lysis, due to a pivotal role of NKp30 and DNAM-1 activating receptors (20, 100), is restricted to iDC undergoing an unfruitful maturation process, characterized by an inadequate MHC class I up-regulation. This mainly impacts on the expression of the non-classical HLA-E molecule, the cognate ligand of the CD94/NKG2A inhibitory receptor. This effect has been interpreted as a negative selection strategy aimed to avoid inappropriate antigen presentation by MHC class I low DCs, which could result in tolerogenic responses. DC that underwent an appropriate maturation program (mature dendritic cells, mDC) are spared from NK-cell mediated attack since they express very high levels of classical MHC class I and significantly up-regulated HLA-E (101, 102). The principal NK cell-derived mediators shaping DC immune-phenotype are represented by TNFα and IFNγ, whose release depends on the synergistic activity of IL-12 and IL-15 produced by pathogen stimulated DC. mDC *de novo* express CCR7, (a phenomenon also occurring during pathogen-driven macrophage polarization toward M1) which confers to these cells the competence for migrating to SLO. In SLO T cell zone, DCs co-localize with NK cells belonging to the CD56^bright^ CD16^low/neg^ subset, which constitutively express CCR7 (101).

The complex interactions among NK cells expressing IL-2Rα (CD25), DC-primed T cells producing IL-2 and mDC result in a conspicuous IFN-γ production by NK cells shaping T cell priming, polarization and adaptive immune responses (101). Whether, in SLO, NK cells may also shape macrophages' functions remains to be elucidated. CCR7^pos^. NK cells can migrate to SLO via HEV since they also express high levels of CD62L. However, a predominant population of CCR7^pos^ CD56^bright^ CD16^neg^ NK cells has been described in seroma fluid, thus depicting afferent lymph as an alternative way for CD56^bright^ NK cells to colonize SLO (8). Interestingly, it has been observed, in seroma, the presence of low numbers of CD56^pos^CD3^neg^ cells expressing CX_3_CR1, KIRs and CD16 molecules, a phenotype usually characterizing classical CD56^dim^ NK cells (8). Although a multiparametric analysis providing information about a possible co-expression of CCR7 was lacking, these data support the hypothesis that, *in vivo*, cytolytic CD56^dim^ NK cells might also migrate to “perturbed” SLO. Along this line, the interaction of CD56^dim^ NK cells with M0 or M2 macrophages polarizing toward M1 upon TLR engagement results in the acquisition of CCR7 and of a fully activated NK cell status characterized by high CD69 and IL2Rα expression, release of large amount of IFN-γ and increased cytolytic activity (83). Thus, in inflammatory conditions, M1-activated CD56^dim^ NK cells, becoming competent for SLO migration thanks to the acquisition of CCR7, might deeply contribute to both immunosurveillance of tumor metastases and control of infected cells. Migration of fully functional CD56^dim^ NK cells to SLO, could be particularly relevant in the context of KIR/KIRL-mismatched haploidentical stem cell transplantations (haplo-HSCT). Indeed, in SLO, NK-mediated killing of recipient mDC and residual T cells might contribute to the low rate of graft vs. host disease (GVHD) and graft rejection documented in this clinical setting (103).

Nevertheless, is there any *in vivo* evidence that CD56^dim^ NK cells might traffic through and leave SLO, thus recirculating via efferent lymph? A few preliminary reports indicate this possibility. Non-reactive LNs or LNs characterized by sinus hyperplasia lack or show low expression of KIR^pos^CD16^pos^ cells. Interestingly, reactive LNs characterized by paracortical/follicular hyperplasia harbor a significant percentage of cells expressing KIR and CD16 and a similar KIR^pos^CD16^pos^ cells enrichment was observed in the efferent lymph (i.e., toracic duct). Several observations, including a difference in the telomerase length, strongly suggest that CD56^bright^ CD16^neg^ KIR^neg^ cells can acquire a KIR^pos^ CD16^pos^ phenotype thanks to the influence of the different pro-inflammatory cytokines present in LNs (28). However, the hypothesis that CD16^pos^ KIR^pos^ NK cells might migrate to and expand in LNs before egressing via efferent lymph cannot be ruled out. In this context, in pathogen-perturbed tissues, CD56^dim^ NK cells interacting with macrophages acquire the competence to SLO migration and, expressing high levels of IL2Rα (83), become highly responsive to IL-2 produced by T cells in the paracortex area of LN.

Pro-inflammatory cytokines are capable of shaping innate and adaptive immune responses also acting on the establishment of the NK cell memory reservoir. Both in mouse and human, it has been described that the CMV-driven onset of memory NK cell populations requires the presence of pro-inflammatory cytokines such as IL-12 and IL-18. Cytokines represent the third signal essential to generate, expand and recall NK cell memory. Signal 1 is represented by receptor-mediated antigen recognition, LY49D in mouse and NKG2C or KIR2DS1 in humans, and signal 2 by co-stimulatory signals, DNAM-1 and CD2 in mouse and human, respectively (15, 104). In addition, cytokines by themselves are capable of generating memory-like NK cells in an antigen–independent setting (14), as NK cells, shortly cultured in the presence of IL-12, IL-15 and IL-18, showed superior IFN-γ and TNF-α production and cytotoxicity in response to tumor targets and conferred more protection to leukemia or melanoma in xenograft mouse models. Thus, full NK cell activation and antigen-dependent or -independent generation of NK cell memory requires cytokines-mediated signals. It should be considered that cytokines also strongly impact on the chemokine receptor repertoire of NK cells. Beside sIL-18 whose capability of inducing CCR7 expression has been discussed above, IL-15 has been shown to down-regulate CX_3_CR1 expression in mouse bone marrow-derived NK cells (10) and in human PB NK cells (12), thus reducing the chemotactic response to CX_3_CL1 ligand (12). IL-12 in association with IL-2 significantly decreased the CXCR3 mRNA and their surface expression in NK cells (9). Additionally, IL-2 alone has been shown to down-regulate the surface expression of CXCR1 as well as of CXCR4 inhibiting NK cell migration in response to CXCL12. On the other hand, IL-2 up-regulated the surface expression of CXCR3 increasing NK cell migration in response to its ligands CXCL9 and CXCL10 (11).

Regarding the migratory properties of memory NK cells, different questions remain unanswered. Do cytokines that drive their onset, impact on their chemokine receptor repertoire contributing to the generation of tissue-resident memory NK cells in various anatomical areas? Does the maintenance of the NK cell memory pool involve tissue-restricted reactivation of resident memory NK cells or do these cells maintain the potential to recirculate? Studies focused on mouse recall response to haptens provided some relevant indications. These studies showed that memory NK cells responsible for the immune response were the CD49a^+^ DX5^neg^ liver resident NK cells, and that the activity of CD18 and P-selectin, molecules involved in trafficking of NK cells, was needed (15). In this scenario, the characterization of human memory NK cells in terms of chemokine receptor expression, before and after cytokine-stimulation, could be particularly relevant.

## Concluding remarks

It has become evident that NK cells are not constituted by a homogeneous population of innate lymphocytes but rather by different subtypes with specific abilities as well as distinct homing properties. Investigating how NK cell subsets distribute in human body has relevance not only for a better understanding of our immune defenses but also for exploiting these cytotoxic cells in therapeutic settings.

It is worth noting that the migratory properties of NK cell subsets are relevant not only for identifying the region in which they should exert their activity. Recent reports indicate that NK cells could acquire specific properties, such as cytotoxicity, only upon their migration to secondary lymphoid organs where the cytokine milieu would induce their further differentiation. At the same time, although the picture of tissue-resident NK cells is still fuzzy, it is conceivable that these subsets of NK cells might locally acquire peculiar properties, such as release of specific soluble factors able to affect their properties but also influence other cells present in the microenvironment. As a matter of fact, NK cells (as well as all other innate lymphoid cells of which they represent the prototype) are more and more emerging as accessory cells able to modulate the functions of neighboring cells, including antigen presenting cells, in the environment in which they are attracted/hosted.

Despite the relevance of these issues, several open questions still remain to be addressed regarding the ability of NK cells to infiltrate and reside in either healthy or pathological tissues. Decades after the discovery of NK cells as lymphocytes able to recognize and kill cancer cells without prior sensitization to them, we still miss a clear and complete depiction of the phenotype and properties of tumor-infiltrating NK cells. Similarly, although a number of studies have now highlighted the relevance of NK cells in the control of viral infections, how these cytotoxic lymphocytes recirculate and/or are retained in infected tissues still remain to be clearly determined, at least in humans.

On the other hand, novel technologies allowing extensive multiparametric analyses, either by mass cytometry or classical flow cytometry, not even conceivable until only a few years ago, might now open new avenues for a comprehensive mapping of tissutal NK cells. The path appears already tracked since we have now, as reported in the present review, a better appreciation of at least some of the molecules and the signaling ruling the homing properties of these innate lymphocytes.

## Author contributions

RC, PC, AD, GF and CB wrote the manuscript. FB, BC and SR read the manuscript and provided critical input.

### Conflict of interest statement

The authors declare that the research was conducted in the absence of any commercial or financial relationships that could be construed as a potential conflict of interest.
